# Supplementary material for: Time cell sequences during delay intervals are not dependent on brain state and do not support hippocampus-dependent working memory
Source: Nat Commun. 2025 Aug 12;16:7470. doi: 10.1038/s41467-025-62498-z (PMC12343883; doi:10.1038/s41467-025-62498-z)
Supplement: Supplementary file 1 — Supplementary Information [file 41467_2025_62498_MOESM1_ESM.pdf]

Figure S1

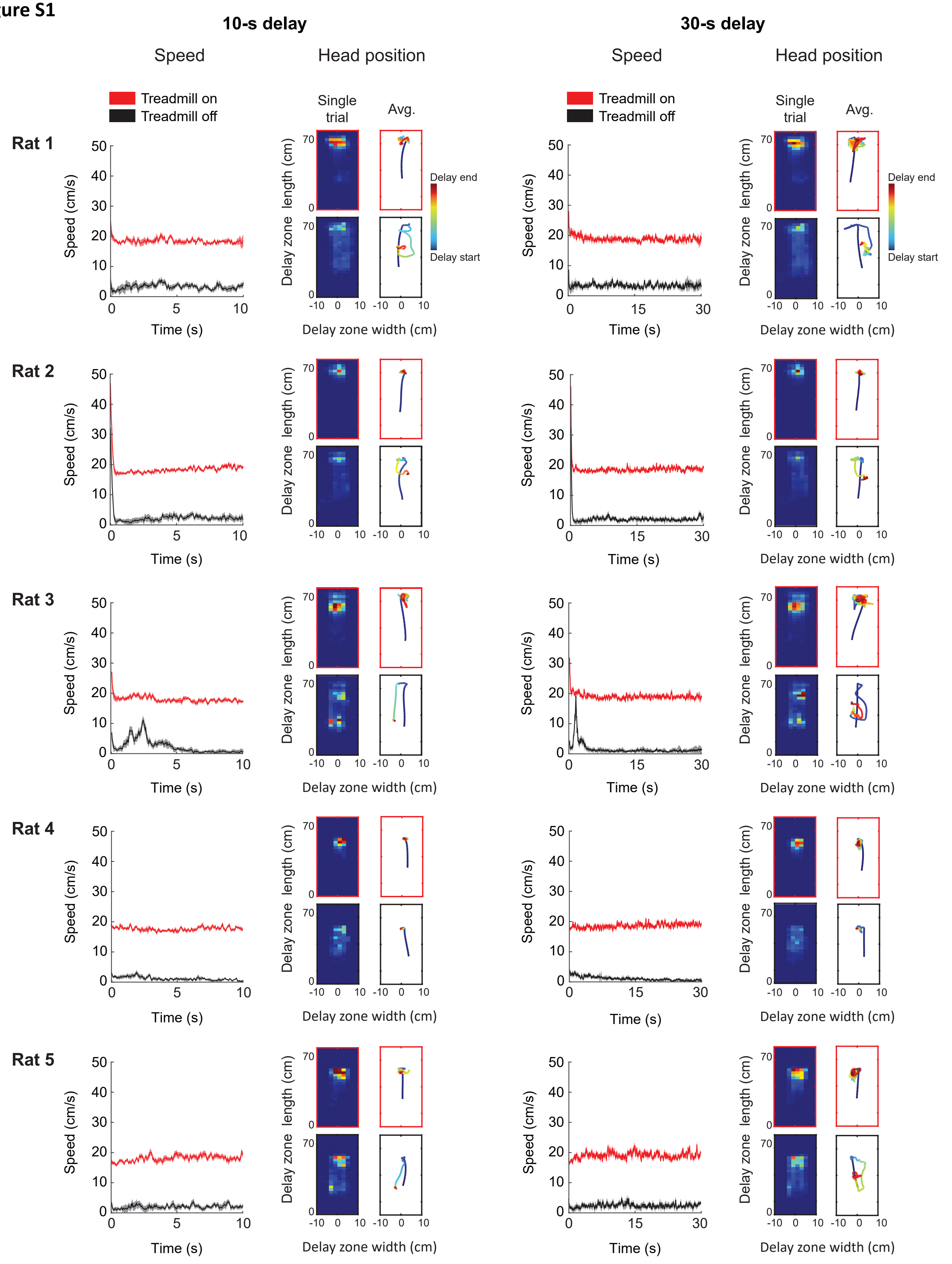

10-s delay
30-s delay
Speed
Head position
Treadmill on
Treadmill off
Single trial
Avg.
Delay end
Delay start
Rat 1
Rat 2
Rat 3
Rat 4
Rat 5
Speed (cm/s)
Time (s)
Delay zone length (cm)
Delay zone width (cm)

Figure S1. Speed and head position during delay intervals, per rat and delay condition. Each row corresponds to delay-period data from a single rat, with 10-s delay trials in the left column and 30-s delay trials in the right column. In each rat/delay panel, the average speed (left) and head position (right) throughout the delay period are plotted separately for treadmill-on (red line or outline) and treadmill-off (black line or outline) trials. Head position: shown for all trials within a condition as an occupancy heat map (red, maximum occupancy time; blue, unvisited locations) and for an example trial (color scale to the right depicts start to end of each trial). The head position remained in a consistent location in front of the barrier during running on the treadmill (i.e., in treadmill-on trials). Therefore, running speed approximately matched the belt speed and was consistent throughout the delay in treadmill-on trials.

Figure S2

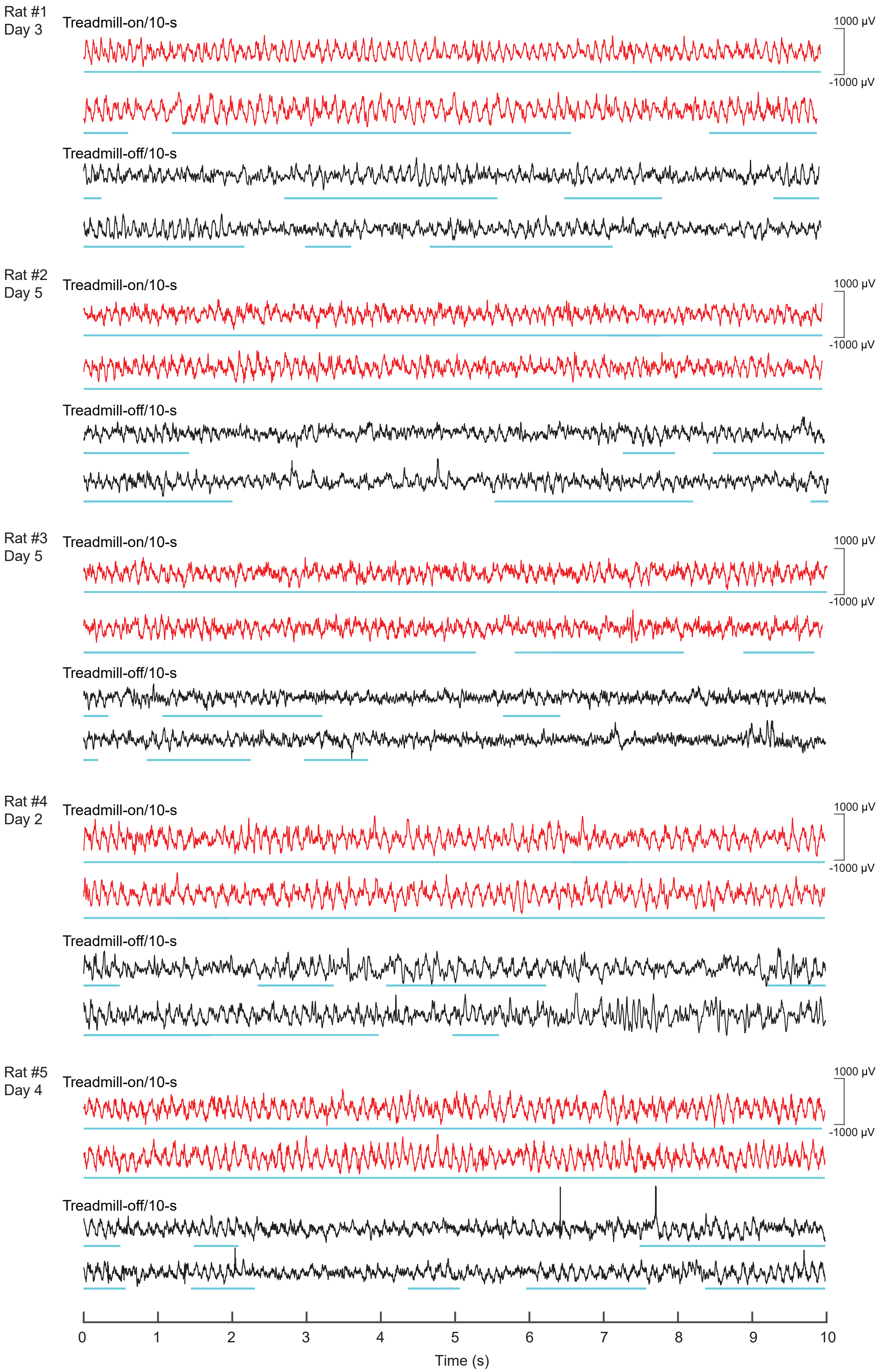

Rat #1
Day 3
Treadmill-on/10-s
Treadmill-off/10-s
Rat #2
Day 5
Treadmill-on/10-s
Treadmill-off/10-s
Rat #3
Day 5
Treadmill-on/10-s
Treadmill-off/10-s
Rat #4
Day 2
Treadmill-on/10-s
Treadmill-off/10-s
Rat #5
Day 4
Treadmill-on/10-s
Treadmill-off/10-s
1000 μV
-1000 μV
0
1
2
3
4
5
6
7
8
9
10
Time (s)

Figure S2. Continuity of theta oscillations during delay intervals with the treadmill on. Per animal (n = 5 rats) examples of unfiltered LFP during the 10-s delay condition with the treadmill either on (red) or off (black). Across treadmill conditions, LFP traces from the same recording site in the same recording session within a day are compared. Blue lines indicate periods of continuous high-amplitude theta oscillations (z-scored theta power > 0.5, see Methods for additional details). The treadmill-on condition was characterized by continuous high-amplitude theta throughout most or all of the 10-s delay, while theta bouts were shorter and more sporadic during the treadmill-off condition (see Figure 1g-j for quantification).

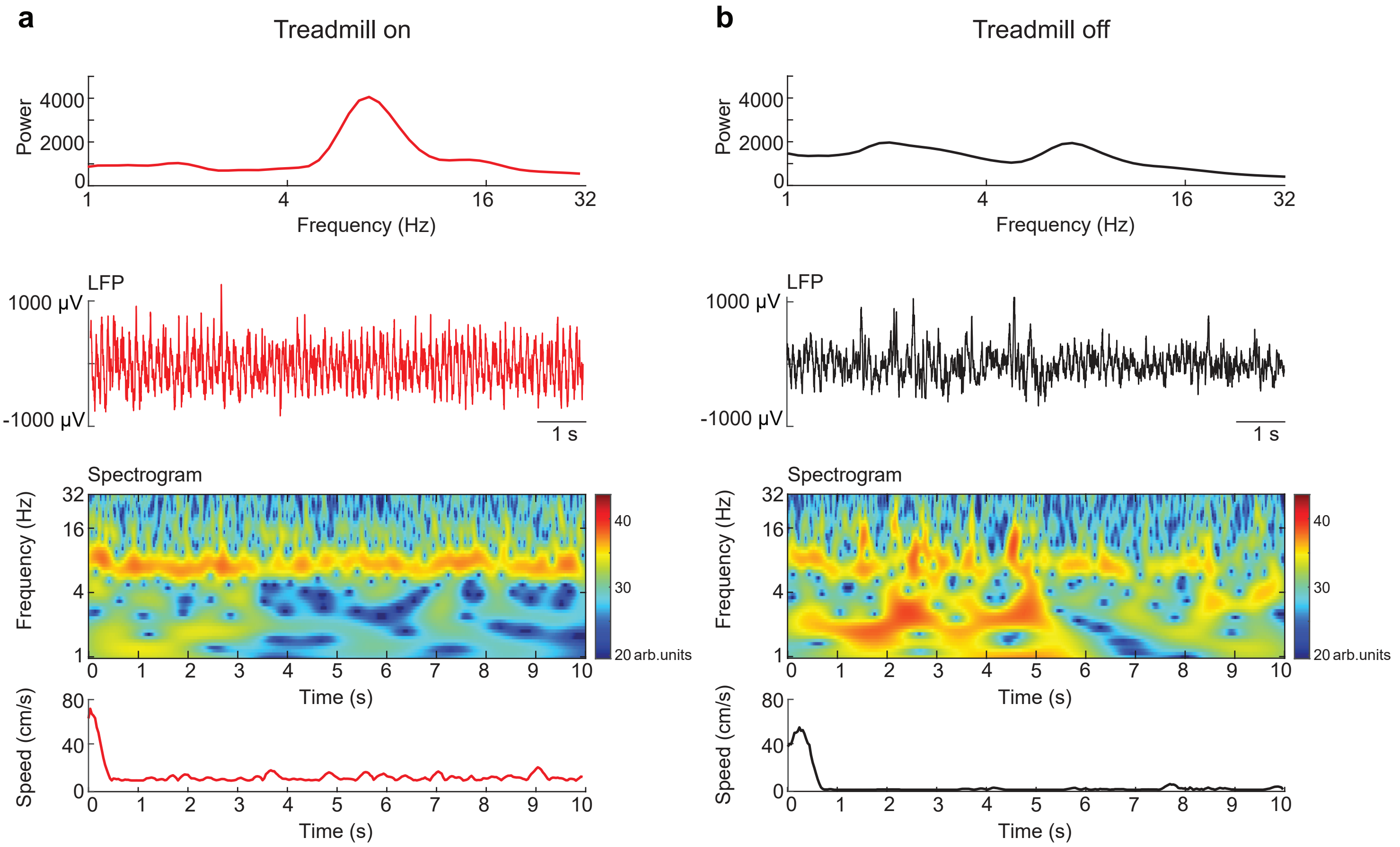

Figure S3. Power spectra and spectrograms confirm differences in theta power between treadmill-on and treadmill-off trials. a, Spectral analysis of the CA1 LFP during an example 10-s delay period in a treadmill-on trial. Top to bottom: power spectral density of the CA1 LFP; raw CA1 LFP throughout the 10-s delay period; spectrogram of time-frequency content of CA1 LFP during the delay period; corresponding animal speed. Note that the rat briefly accelerates when running/jumping onto the treadmill. **b**, Spectral analysis of the CA1 LFP during an example 10-s delay period in a treadmill-off trial, taken from the same recording site and session as the LFP data in the treadmill-on trial (shown in a). Panels ordered as in a.

Figure S4

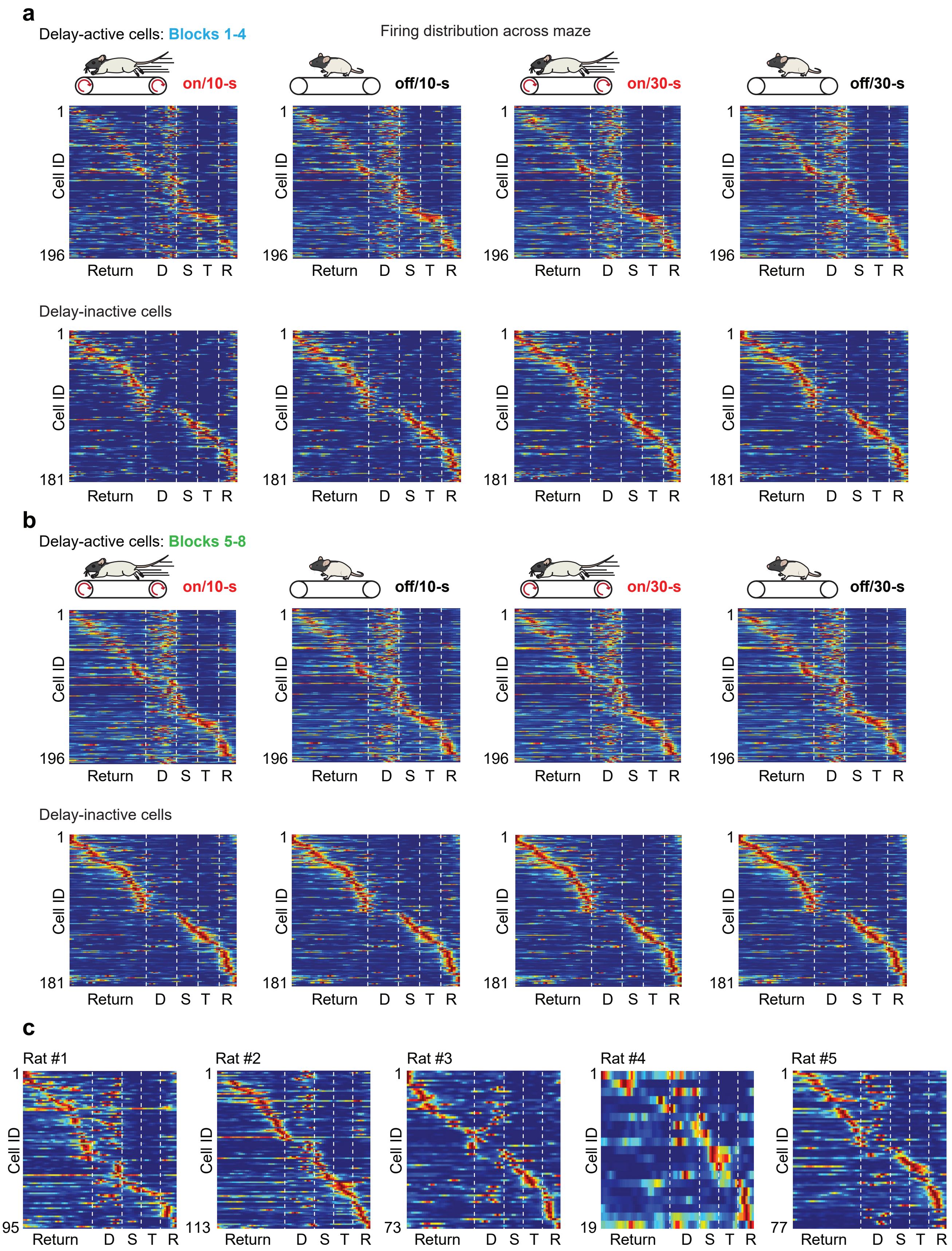
a
Delay-active cells: Blocks 1-4
Firing distribution across maze
on/10-s
off/10-s
on/30-s
off/30-s
Cell ID
1
196
Return D S T R
Delay-inactive cells
181
b
Delay-active cells: Blocks 5-8
c
Rat #1
Rat #2
Rat #3
Rat #4
Rat #5
95
113
73
19
77

Figure S4. Spatial distribution of the firing locations of delay-active and delay-inactive cells. Spatial distribution of the cells' firing locations on the maze during the first and second half of behavioral trials within a day. **a**, Top: blocks 1-4 (10 trials per block; see Figure 1a). Spatial distribution of delay active cells on the maze in each treadmill/delay condition (number of delay-active cells across all 4 conditions: n = 196). Bottom: spatial distribution of delay-inactive cells on the maze in each treadmill/delay condition (number of delay-inactive cells across all 4 conditions: n = 181). Cells are sorted by the peak firing location across the average over the four conditions. **b**, Same cells as shown in a, but during blocks 5-8. Data are plotted and sorted as in a. **c**, Spatial distribution of all putative CA1 pyramidal cells from each rat. Data are from all trials within a session (blocks 1-8), and cells are sorted by their peak firing location. Blue, 0 Hz; red, each cell's maximum rate within a condition; D, delay zone; S, stem of maze; T, T zone; R, reward zone.

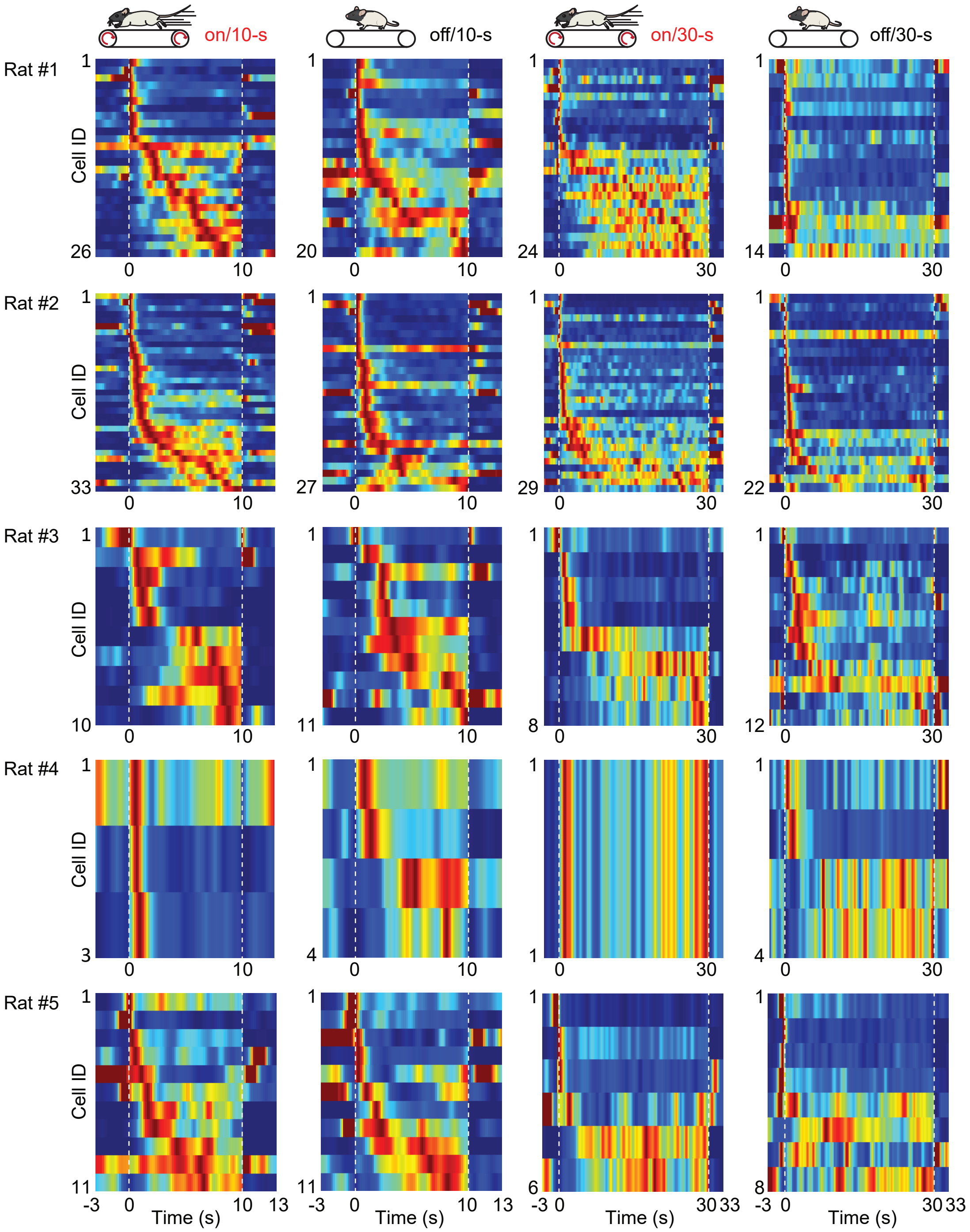

Figure S5. Per animal time cell distribution during each delay condition. For each animal the principal cells that met time cell classification criteria are sorted by their peak firing time within each delay condition. Blue, 0 Hz; red, each cell's maximum rate within a condition. The mean fraction of time cells compared to all putative pyramidal cells (n = 5 rats; n = 95, 113, 73, 19 and 77 pyramidal cells for rats #1-#5) was 20.1% ± 3.38, 15.0% ± 4.37, 19.1% ± 1.87 and 16.4% ± 1.87 in the on/10-s, on/30-s, off/10-s and off/30-s conditions. See Table S3 for the mean fraction of cells with other classification criteria, such as delay-active cells, time-limited cells and persistently active cells.

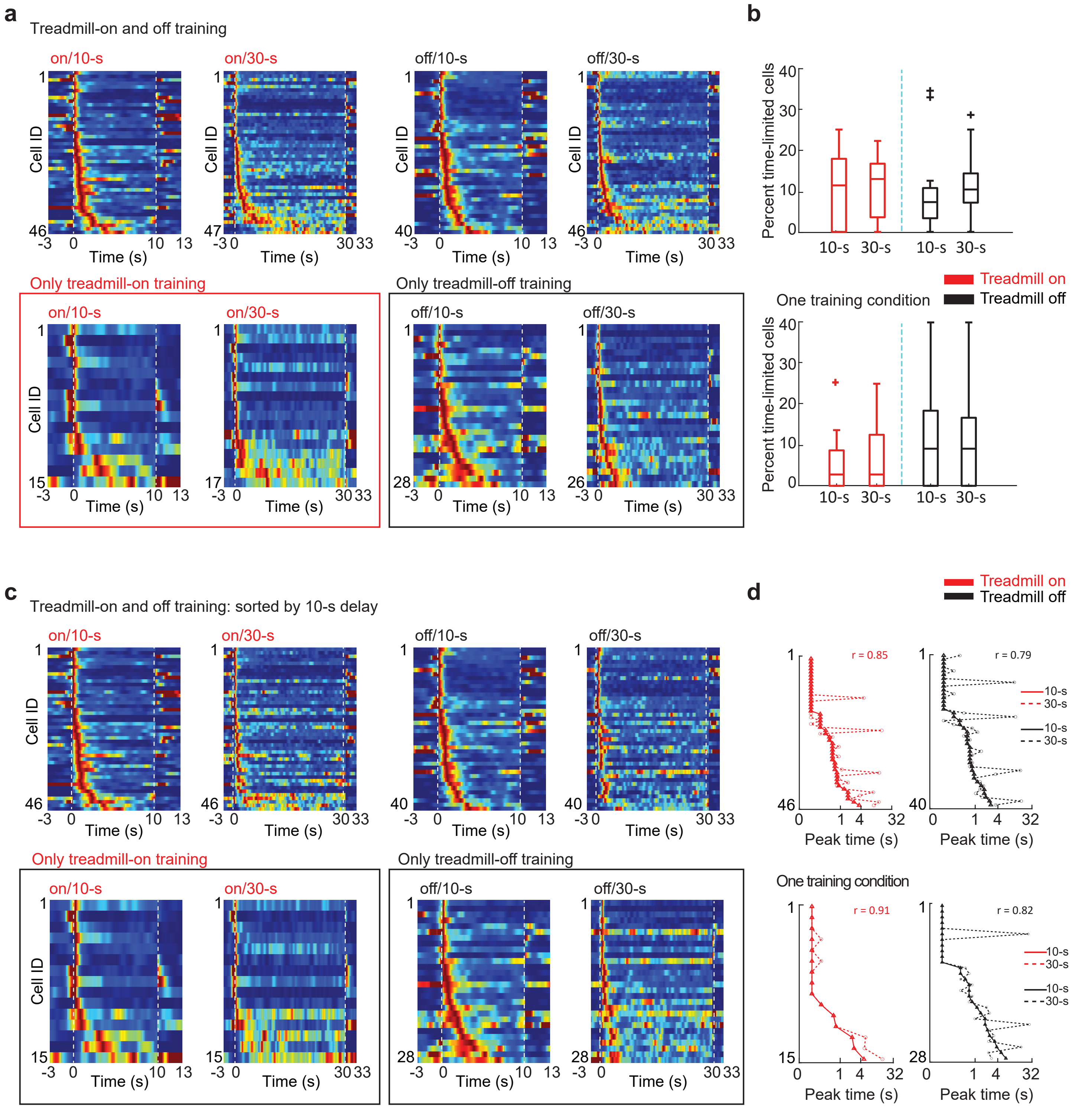

Figure S6. Time-limited cells from rats trained in different treadmill conditions. a, Top: normalized firing rates (blue: 0 Hz; red: each cell's maximum rate within a condition) of time-limited cells from rats trained with treadmill-on and treadmill-off conditions (on/10-s, on/30-s, off/10-s or off/30-s, in pseudorandomized order within each session). Each line is a cell, and cells are ordered by the peak time within each condition. Bottom: normalized firing rates of time-limited cells from rats trained only in treadmill-on conditions (left) or treadmill-off conditions (right). Each line is a cell, and cells are ordered by the peak time within each condition. **b**, The fraction of time-limited cells in each treadmill/delay condition. Top: rats with training in both treadmill conditions

Figure S6 continued...
(on/10-s: 11.18% ± 2.19, on/30-s: 10.74% ± 1.89, off/10-s: 9.09% ± 2.40, off/30-s: 12.24% ± 1.92, mean ± SEM, n = 18 sessions; $F(3,68) = 0.41$, $p = 0.75$, ANOVA). Bottom: rats with training in either the treadmill-on or treadmill-off condition (treadmill on, 10-s: 6.17% ± 2.21, 30-s: 6.74% ± 2.16; treadmill off, 10-s: 10.76% ± 2.07, 30-s: 10.23% ± 2.24). For treadmill-on conditions, there was a lower proportion of time-limited cells in the group with only treadmill-on training compared to the group with combined training (Training: $F(1,64) = 4.82$, $p = 0.032$, Duration: $F(1,64) = 0.001$, $p = 0.98$, Training*Duration: $F(1,64) = 0.061$, $p = 0.81$, two-way ANOVA). For treadmill-off conditions, there was no significant difference between the group with only treadmill-off training and the combined training group (Training: $F(1,82) = 0.006$, $p = 0.94$; Duration: $F(1,82) = 0.37$, $p = 0.55$; Training*Duration: $F(1,82) = 0.72$, $p = 0.40$, two-way ANOVA). **c**, Normalized firing rates (red: maximum rate, blue: 0 Hz) of time-limited cells that were identified in the 10-s delay condition. Cells in both the 10-s delay and 30-s delay panels are ordered by the peak time in the 10-s delay condition. Top: rats trained in both treadmill conditions. Bottom: rats trained in only one treadmill condition. **d**, The peak firing time was correlated between the 10-s and 30-s delay conditions. Top: rats trained in both conditions (10-s vs 30-s, treadmill-on condition: Spearman's r = 0.85, $p = 1.3 \times 10^{-14}$, n = 46 cells; treadmill-off condition: Spearman's r = 0.79, $p = 5.9 \times 10^{-10}$, n = 40 cells). Bottom: rats trained in only one treadmill condition, with the treadmill either on in all trials or off in all trials (treadmill-on: Spearman's r = 0.91, $p = 2.1 \times 10^{-6}$, n = 15 cells; treadmill off: Spearman's r = 0.82, $p = 1.0 \times 10^{-7}$, n = 28 cells). All statistical tests are two-sided without adjustments for multiple comparisons. Box plots: central line, edges, whiskers and plus signs indicate median, the 25th/75th percentile, maximum/minimum and outliers. Source data are provided as a Source Data file.

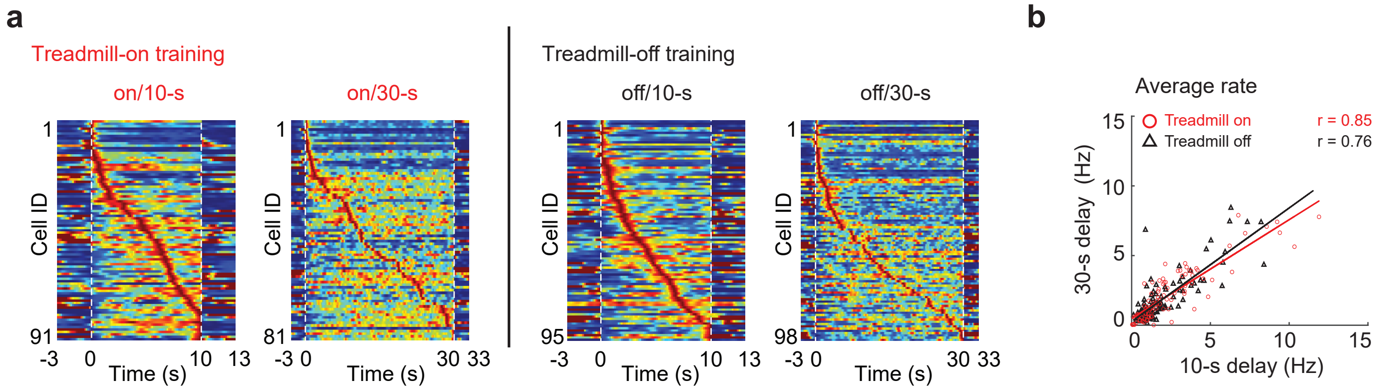

Figure S7. Delay-active cells from rats trained on only one of the treadmill-on or off conditions. Delay-active cells, sorted by their peak firing time within the delay period. Blue, 0 Hz; red, each cell's maximum rate within a condition. **a**, Delay-active cells, (left) from rats trained only in the treadmill-on condition (n = 4 rats, delay-active cells, on/10-s: n = 91, on/30-s: n = 81; or (right) in only the treadmill-off condition (n = 4 rats, delay-active cells, off/10-s: n = 95, off/30-s: n = 98). **b**, Average firing rates were strongly correlated across 10-s and 30-s delay conditions (treadmill on: Spearman's r = 0.85, $p < 0.0001$, n = 95 cells from 4 rats, cells included if active in at least one of the two delay conditions; treadmill off: Spearman's r = 0.76, $p < 0.0001$, n = 98 cells from 4 rats, linear regression) regardless of whether the animal ran during the entire delay or not. All statistical tests are two-sided without adjustments for multiple comparisons. Source data are provided as a Source Data file.

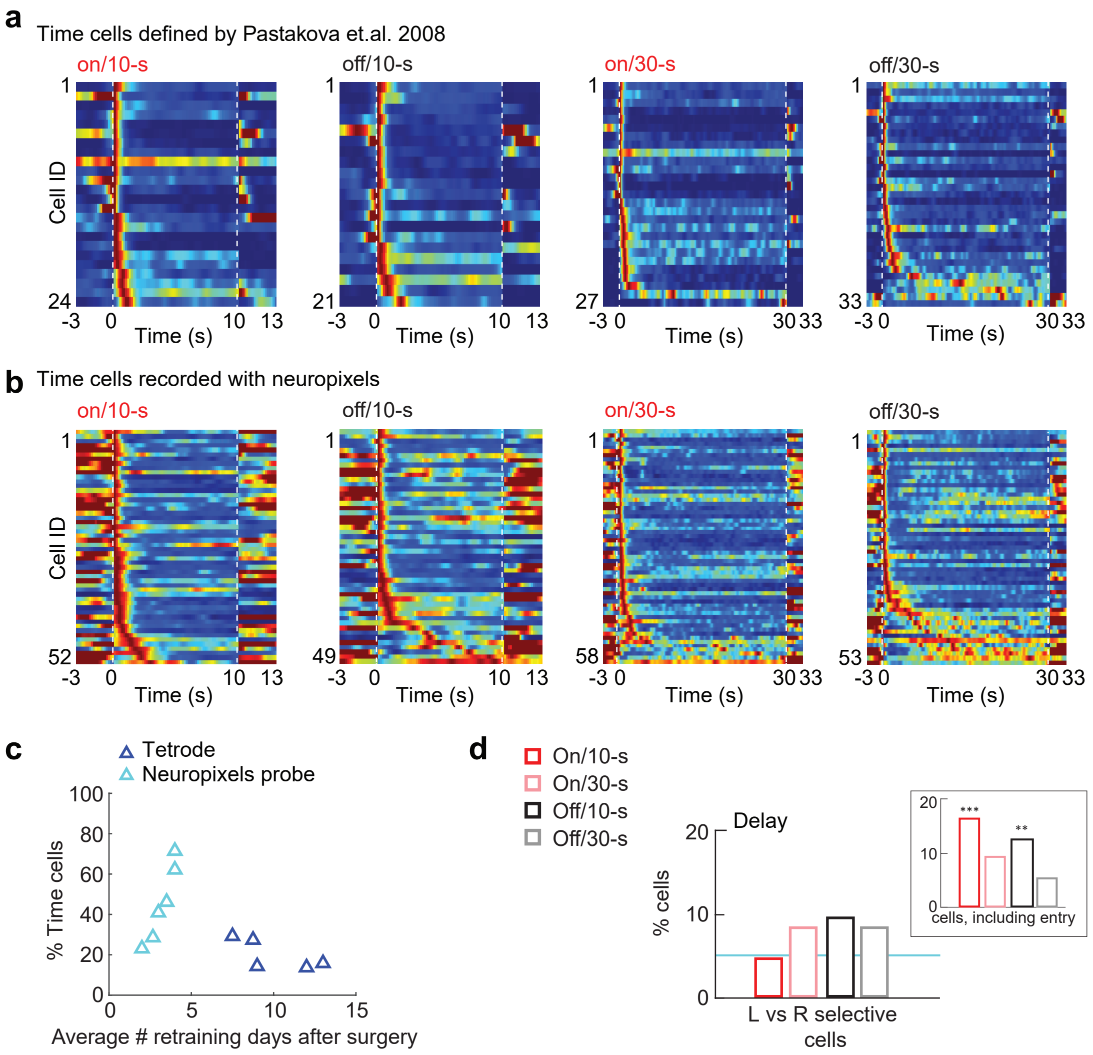

Figure S8. Time cells classified by using a previously published method or after shorter training durations.
a, Normalized firing rates (blue, 0 Hz; red: each cell's maximum rate within a condition) of time cells, using the definition provided by ref. 8. Each line is a cell, and cells are ordered by the peak time within each condition. The fraction of time cells classified by the method in ref. 8 was lower compared to the fraction classified by our shuffle-based method (method from ref. 8, on/10-s: 4.94% ± 1.90; on/30-s: 5.80% ± 1.91; off/10-s: 5.35% ± 1.11; off/30-s: 7.71% ± 1.80, mean ± SEM, n = 5 rats; shuffle-based method, see also Figure 4a: 20.1% ± 3.38, 15.0% ± 4.37, 19.1% ± 1.87 and 16.4% ± 1.87, mean ± SEM, n = 5 rats; $F(1,39) = 44.32$, $p = 2*10^{-7}$, ANOVA: two-factor with replication). **b**, Normalized firing rates (red: maximum rate, blue: 0 Hz) of time cells, recorded after fewer post-surgery retraining days (< 5 days, Neuropixels recordings, on/10-s: 45.40% ± 9.38; on/30-s: 38.13% ± 12.21; off/10-s: 54.55% ± 8.84; off/30-s: 45.77% ± 8.47; mean ± SEM, n = 6 rats; > 5 days, tetrode recordings: 20.1% ± 3.38, 15.0% ± 4.37, 19.1% ± 1.87 and 16.4% ± 1.87, mean ± SEM, n = 5 rats; $F(1,41) = 88.07$, $p = 6*10^{-11}$, ANOVA: two-factor with replication). **c**, The proportion of time cells per rat is plotted against the number of post-surgery training days prior to the current recording session (dark blue triangles, tetrode recordings, n = 5 rats; light blue triangles, Neuropixels probe recordings, n = 6 rats). When multiple recording days were averaged for a rat, the training days value was averaged across included days. **d**, Proportion of turn-selective cells in the delay zone. No significant turn selectivity was observed during the delay period (cells: on/10-s, p = 0.599; on/30-s, p = 0.122; off/10-s, p = 0.056; off/30-s, p = 0.122, n = 83 cells, chance level = 5%, blue line) except when the analysis segment was expanded to include the entry into the delay zone (i.e., with the head, but not the body inside the delay zone) (inset: cells, on/10-s, p < 0.001; on/30-s, p = 0.062; off/10-s, p = 0.003; off/30-s, p = 0.421, n = 85 cells). All statistical tests are two-sided without adjustments for multiple comparisons. Source data are provided as a Source Data file.

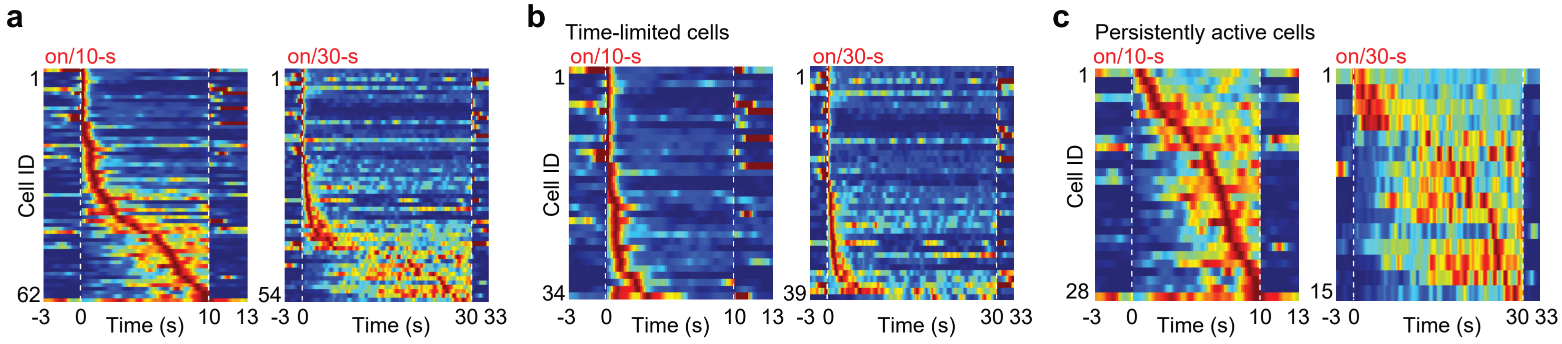

Figure S9. When using only treadmill-on trials with near-continuous theta activity, time cell activity patterns were consistent with data that included all treadmill-on trials. a, Normalized firing rates (blue, 0 Hz; red: each cell's maximum rate within a condition) of time cells, with analysis restricted to treadmill-on trials with high-amplitude theta throughout at least 80% of the delay interval. Each line is a cell, and cells are ordered by the peak time within each condition. The fraction of time cells in high-theta trials did not differ from the fraction in all treadmill-on trials (high-theta trials: on/10-s: 15.36% ± 3.54; on/30-s: 13.03% ± 3.44; all trials: on/10-s: 20.07% ± 3.38; on/30-s: 14.99% ± 4.37; mean ± SEM, n = 5 rats; $F(1,19) = 0.81$, $p = 0.382$, ANOVA: two-factor with replication). **b**, Normalized firing rates (red: maximum rate, blue: 0 Hz) of time-limited cells, with analysis restricted to trials with high-amplitude theta. Each line is a cell, and cells are ordered by the peak time within each condition. The fraction of time-limited cells in high-theta trials did not differ from the fraction in all treadmill-on trials (high-theta trials, on/10-s: 8.64% ± 2.26; on/30-s: 8.71% ± 2.97; all trials, on/10-s: 11.25% ± 2.44; on/30-s: 10.51% ± 2.98; mean ± SEM, n = 5 rats; $F(1,19) = 0.68$, $p = 0.423$, ANOVA: two-factor with replication). **c**. Normalized firing rates (red: maximum rate, blue: 0 Hz) of persistently active cells, with analysis restricted to trials with high-amplitude theta. Each line is a cell, and cells are ordered by the peak time within each condition. The fraction of persistently active cells in high-theta trials did not differ from the fraction in all treadmill-on trials (high-theta trials, on/10-s: 6.72% ± 1.49; on/30-s: 4.32% ± 1.00; all trials, on/10-s: 8.82% ± 1.27; on/30-s: 4.48% ± 1.61; mean ± SEM, n = 5 rats; $F(1,19) = 0.68$, $p = 0.418$, ANOVA: two-factor with replication).

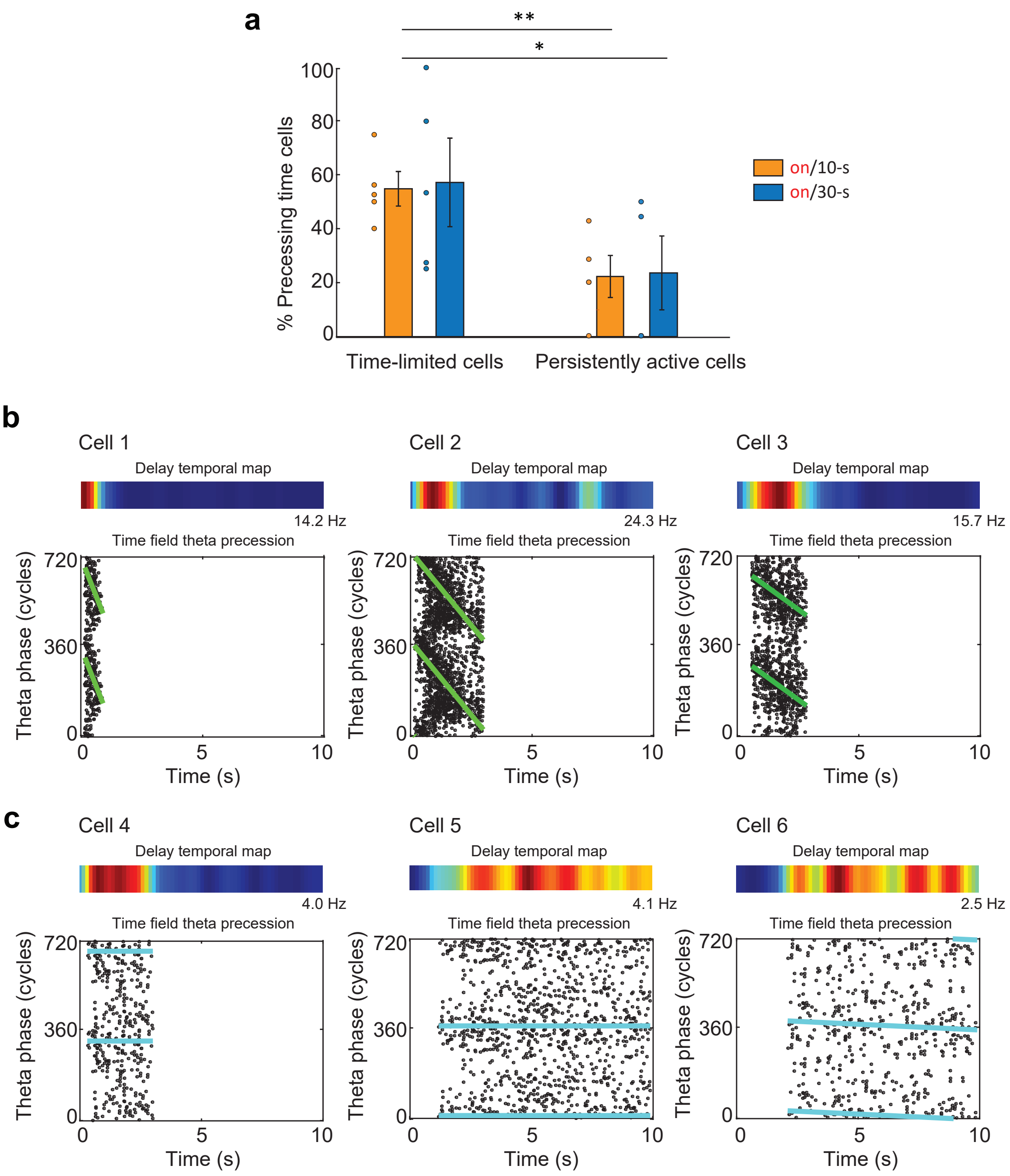

Figure S10. Theta phase precession of time cells. a, Percentage of phase precessing cells within the time-limited cell population and within the persistently active cell population (time-limited cells, on/10 s: 54.78% ± 6.41; on/30-s: 57.12% ± 16.39; persistently active cells, on/10-s: 22.29% ± 7.79; on/30-s: 23.61% ± 13.68; mean ± SEM, n = 5 rats; $F(3,16) = 3.90$, $p = 0.029$, ANOVA). Filled circles are data from individual rats. Analysis was performed for delay intervals of all treadmill-on trials. **b**, Three example time cells with significant theta phase precession during the 10-s delay interval (circular-linear regression, $p < 0.05$). Top: color-coded firing rate during the on/10-s delay (red, maximum rate; blue, 0 Hz). Bottom: phase-versus-time raster plot. Green regression lines in the plot indicate significant phase precession (circular-linear regression, $p < 0.05$). **c**, Three example time cells that did not exhibit theta phase precession. Top: color-coded firing rate during the on/10-s delay (red, maximum rate; blue, 0 Hz). Bottom: phase-versus-time raster plot. Blue regression lines indicate the lack of phase precession (i.e., phase precession slopes are not significant). * $p < 0.05$, ** $p < 0.01$. Source data are provided as a Source Data file.

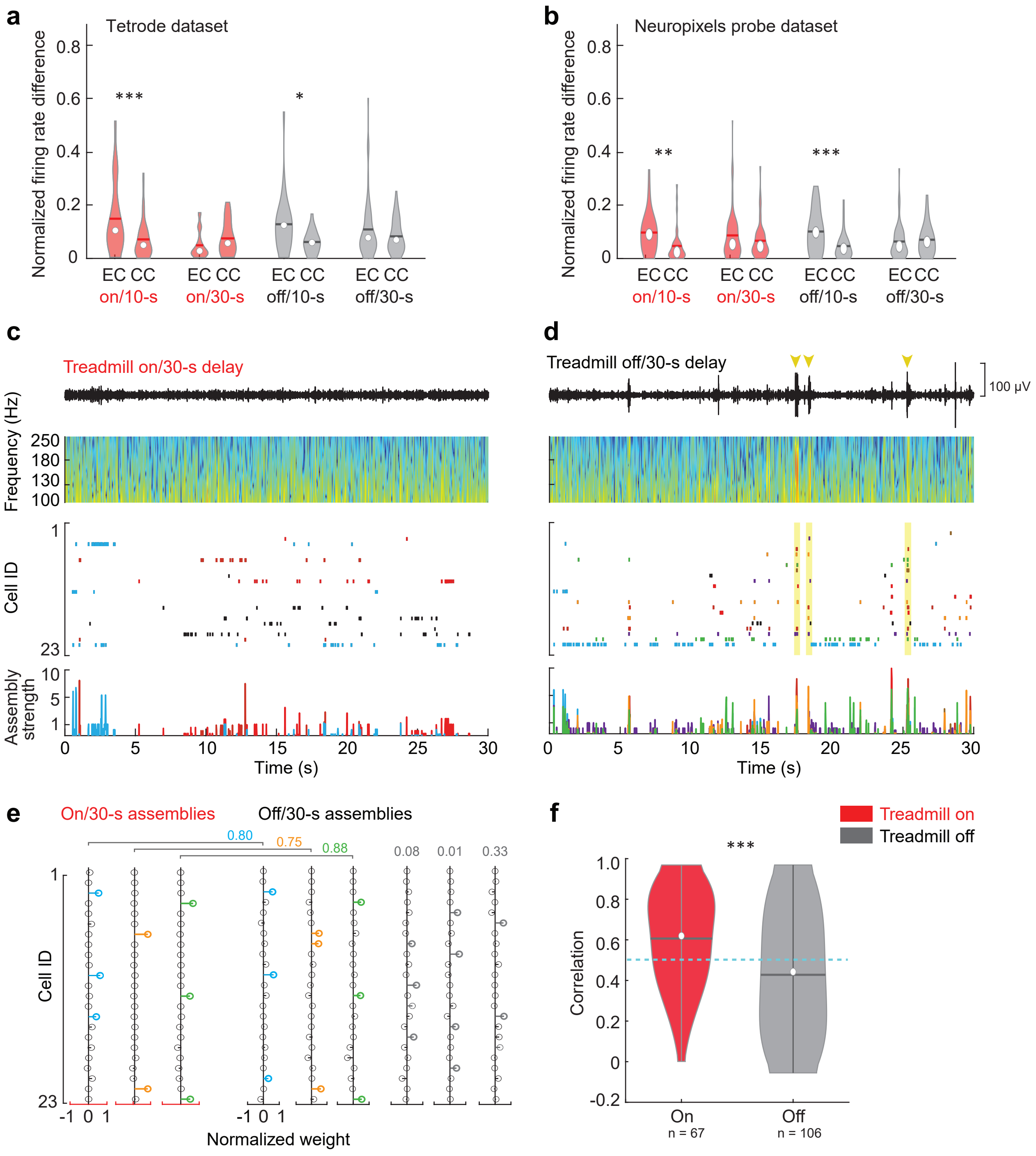

Figure S11. Hippocampal population dynamics across treadmill conditions. a and **b**, Hippocampal pyramidal neurons exhibited higher differences in firing rates between error and correct trial pairs (EC) than between pairs of correct trials (CC), but only during 10-s delay intervals and not 30-s intervals. For each cell, rate differences were averaged over all possible EC pairs and over an equal number of CC pairs. Statistical comparisons were performed by using the cells' average values (data from tetrode recordings, on/10-s: p = 0.0004, n = 50 cells, on/30-s: p = 0.1468, n = 25 cells; off/10-s: p = 0.0033, n = 27 cells; off/30-s: p = 0.0614, n = 68 cells; data from the Neuropixels recordings, on/10-s: p = 0.0019, n = 36 cells; on/30-s: p = 0.2481, n = 51 cells; off/10-s: p = 0.0005, n = 39 cells; p = 0.5341, n = 58 cells; t-tests). Violin plots: Horizontal line, mean; white dot, median; grey outlines, distribution. **c**, Hippocampal population activity during an example 30-s delay period with the treadmill on. Top to

Figure S11 continued...
bottom: Bandpass-filtered LFP (100-250 Hz); spectrogram of CA1 LFP; spike raster plot from all active putative CA1 pyramidal neurons (color-coded according to the simultaneously active neuronal assembly in which they participate) and assembly reactivation strength over time. **d**, Hippocampal population activity during an example 30-s delay period with the treadmill off. Panels are ordered as in c, and the data are from the same animal, tetrode, and recording session as in c. Identified sharp wave ripple events (see Methods) are identified by yellow arrows in the LFP trace and the corresponding spike activity is highlighted in yellow in the spike raster plot. **e**, Assemblies detected during the treadmill-on and treadmill-off delays were compared for similarity. For three example cell assemblies, the highest cosine similarity between the assembly and any of the assemblies from the opposite treadmill condition (i.e., on vs. off) is shown (correlation values at the top of the panel). For illustration, cell assemblies with high similarity scores are shown with matching colors (although they were otherwise detected and analyzed separately), and assemblies without a high similarity score are shown in gray. **f**, Distribution of similarity scores across treadmill conditions. Scores for all assemblies in detected in treadmill-on (0.61 ± 0.03, mean ± SEM, $n = 67$) were higher than scores for assemblies detected in treadmill-off (0.43 ± 0.03, mean ± SEM, $n = 106$, $p = 0.0007$, Kolmogorov-Smirnov test; grey line, mean, white dot, median, grey outlines, distribution), indicating that treadmill-on assemblies were more likely to have a corresponding assembly in treadmill-off than vice versa. Blue dashed line corresponds to a threshold of 0.5, which was chosen as the separation point for an assembly to be considered shared between treadmill-on and treadmill-off conditions. All statistical tests are two-sided without adjustments for multiple comparisons. * $p < 0.05$, ** $p < 0.01$, *** $p < 0.001$. Source data are provided as a Source Data file.

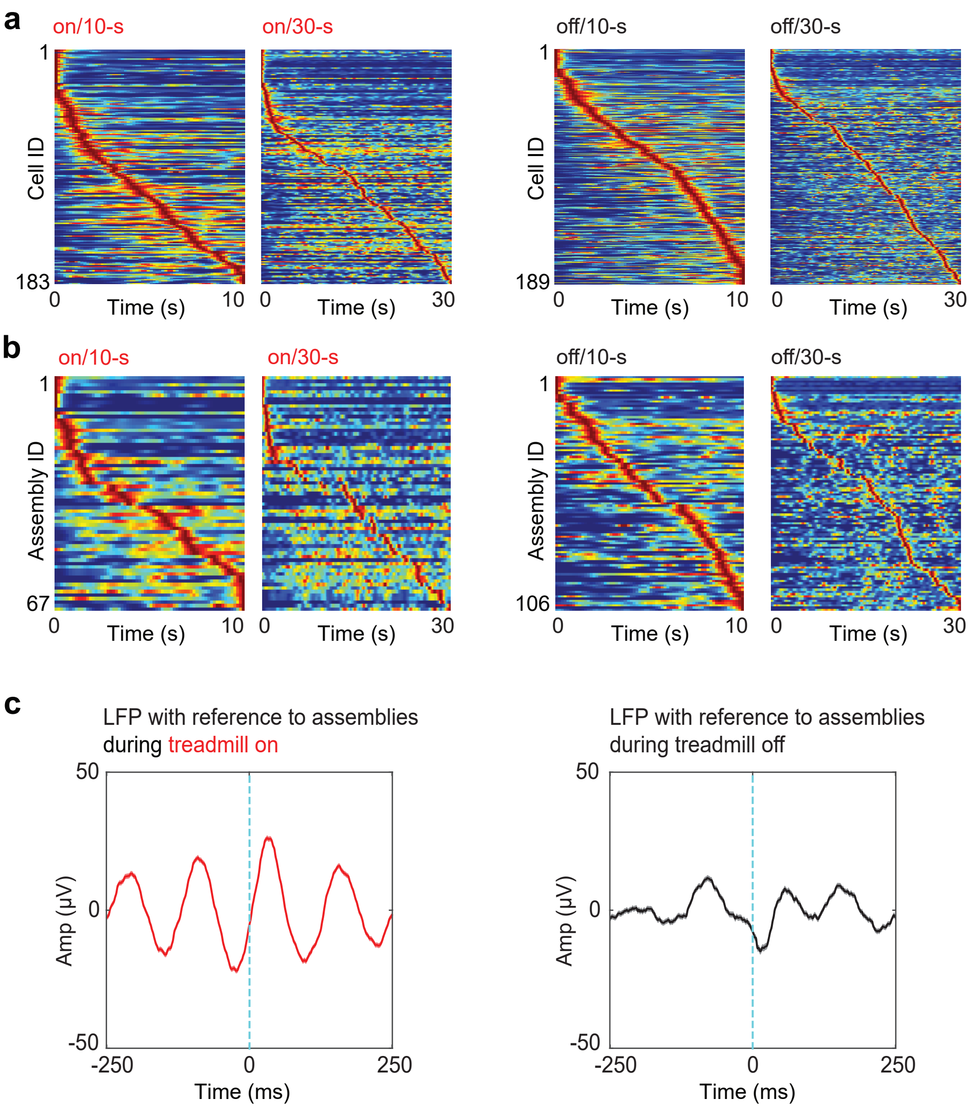

Figure S12. Assembly activity during delay intervals with ongoing theta oscillations (i.e., treadmill-on) showed theta phase preference. a, Normalized firing rates (blue: 0 Hz, red: each cell's maximum rate within a condition) of delay-active cells. Cells that were either delay-active in the 10-s or 30-s delay are included. Each line is a cell, and cells are ordered by the peak time within each condition. Left: treadmill-on conditions. Right: treadmill-off conditions. **b**, Normalized incidence rates (blue: 0 Hz, red: each assembly's maximum rate within a condition) of delay-active cell assemblies. Each line is an assembly, and assemblies are ordered by the peak activity time within each condition. Left: treadmill-on conditions. Right: treadmill-off conditions. **c**, Average peri-event local fields potentials, centered on assembly events in the treadmill-on (left) and treadmill-off (right) condition.

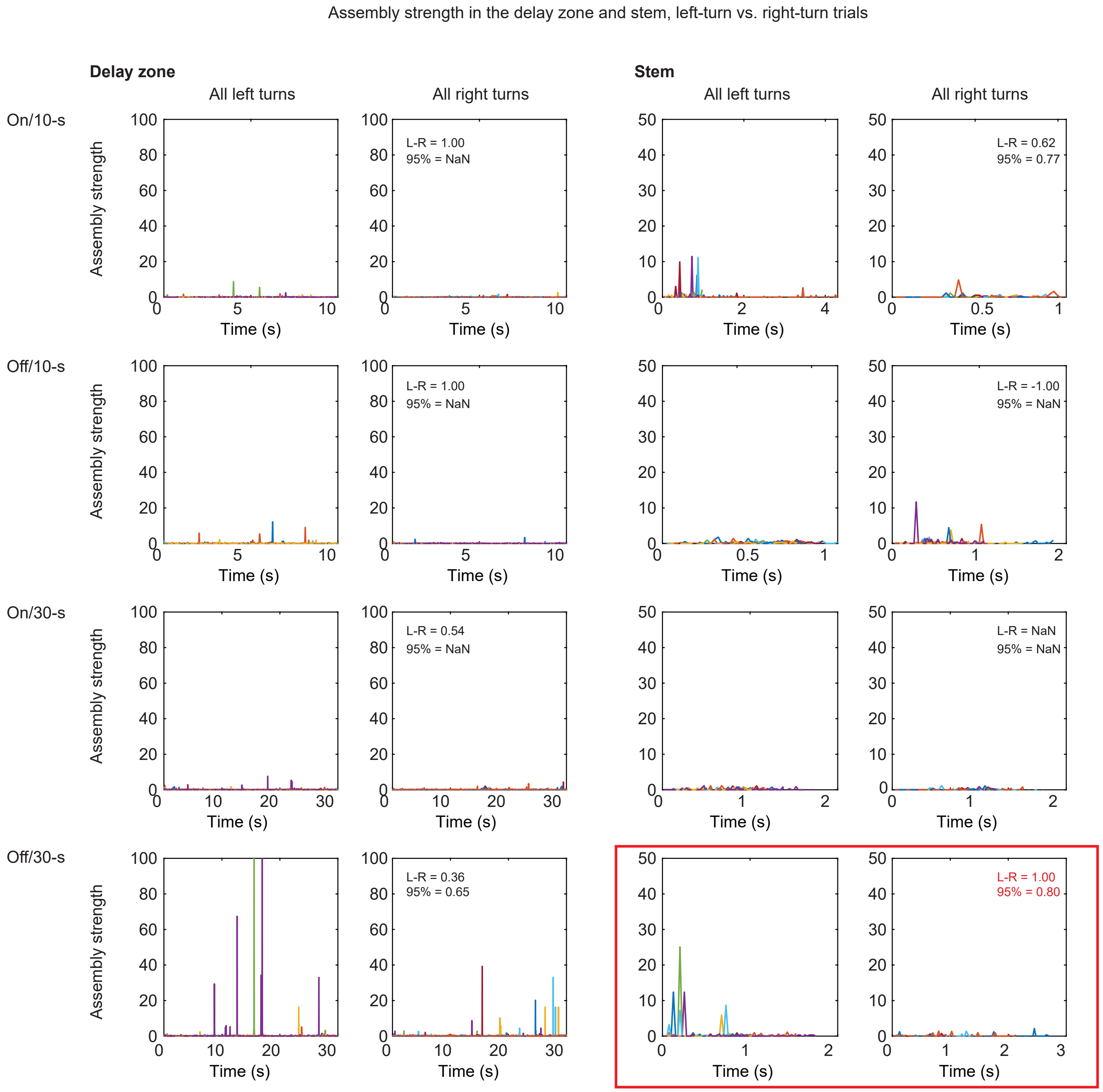

Figure S13. Example cell assembly that was turn-selective in the stem. The first two columns display the strength of one example assembly in the delay area, and the last two columns display the strength of the same assembly in the stem, with different colors representing different trials. In the treadmill-off/30-s delay condition, the assembly was active during the delay, but without turn selectivity. Immediately after exiting the delay, the assembly strength was highly turn-selective in the stem. For each left vs. right comparison, the actual difference in strength is indicated on top and the 95th percentile threshold is indicated below. NaN indicates an empty value, when an assembly was active in fewer than 4 trials. Red box highlights condition with significant turn-selective assembly strength.

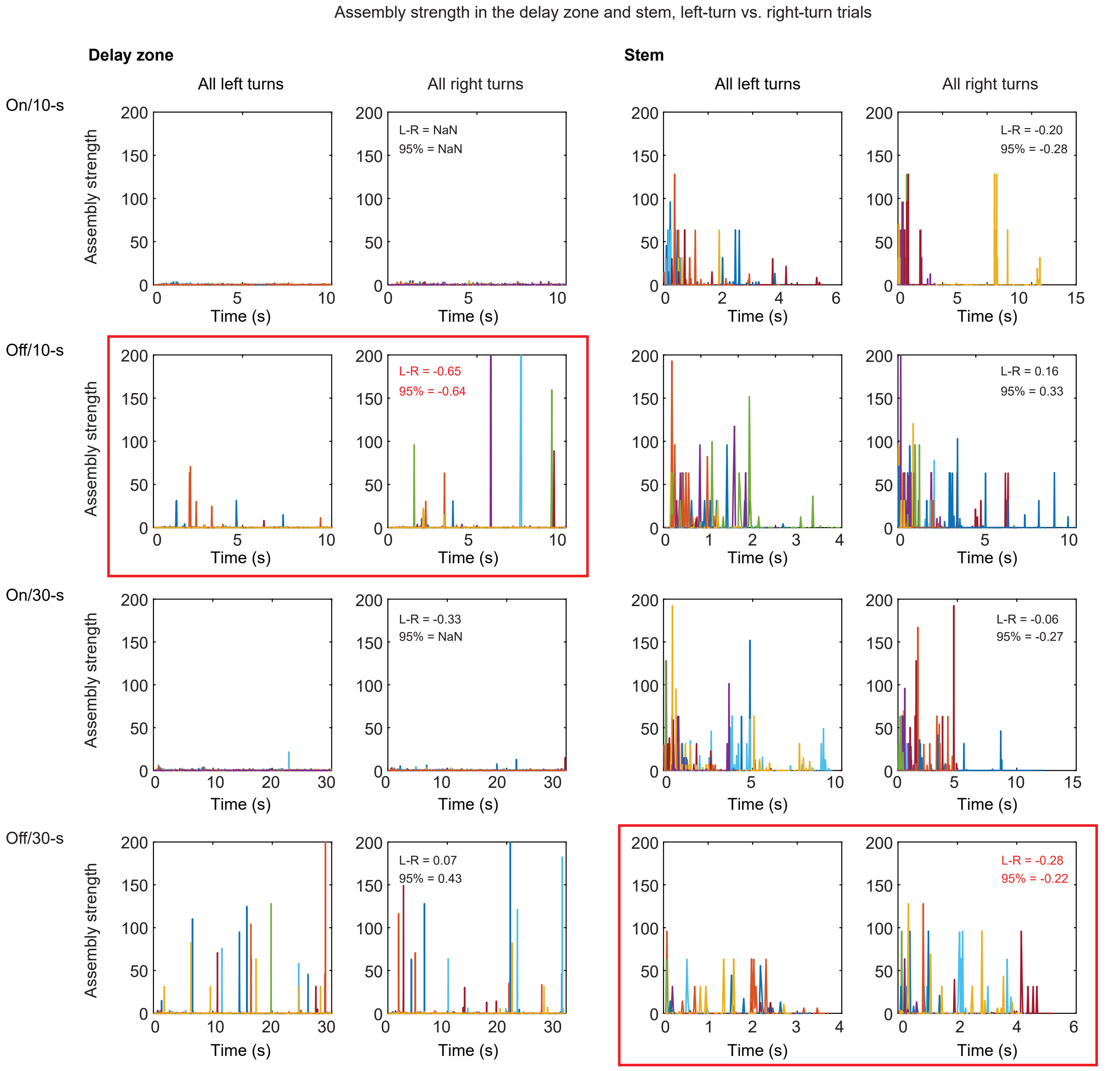

Figure S14. Example cell assembly that was turn-selective in the delay and in the stem. The first two columns display the strength of one example assembly in the delay area, and the last two columns display the strength of the same assembly in the stem, with different colors representing different trials. In the treadmill-off/10-s delay condition, the assembly was turn-selective during the delay. In the treadmill-off/30-s delay condition, the assembly was turn-selective in the stem. For each left vs. right comparison, the actual difference in strength is indicated on top and the 95th percentile threshold is indicated below. NaN indicates an empty value, when an assembly was active in fewer than 4 trials. Red boxes highlight conditions with significant turn-selective assembly strength. Note that a low fraction of cell assemblies was differentially active in the delay, but that the fraction did not exceed chance levels (blue line; see Figure 7g). See Table S4 for statistics.

Table S1. Statistics for correlation values, comparisons of normalized firing rates between pairs of 5-s intervals.

Treadmill on

Correlation between normalized rates, across pairs of 5-s segments as shown in Figure 3E

	10-s delay 0-5 s vs. 30-s delay 0-5 s	10-s delay 0-5 s vs. 10-s delay 5-10 s	30-s delay 0-5 s vs. 30-s delay 5-10 s	30-s delay 5-10 s vs. 30-s delay 10-15 s	30-s delay 5-10 vs 30-s delay 15-20 s	30-s delay 5-10 s vs. 30-s delay 20-25 s	30-s delay 5-10 s vs. 30-s delay 25-30 s
r value	0.61	-0.49	-0.30	0.51	0.48	0.21	0.24
p value	7.81E-10	2.15E-06	0.0059	6.78E-07	4.15E-06	0.0555	0.0293

Treadmill off

Correlation between normalized rates, across pairs of 5-s segments as shown in Figure S2B

	10-s delay 0-5 s vs. 30-s delay 0-5 s	10-s delay 0-5 s vs. 10-s delay 5-10 s	30-s delay 0-5 s vs. 30-s delay 5-10 s	30-s delay 5-10 s vs. 30-s delay 10-15 s	30-s delay 5-10 vs 30-s delay 15-20 s	30-s delay 5-10 s vs. 30-s delay 20-25 s	30-s delay 5-10 s vs. 30-s delay 25-30 s
r value	0.68	-0.26	-0.46	0.43	0.32	0.2	0.07
p value	3.46E-11	2.54E-02	5.05E-05	1.33E-04	5.90E-03	0.0952	0.5784

Treadmill on

Correlation between normalized rates, across pairs of adjacent 5-s segments in 30-s delay interval

	30-s delay 0-5 s vs. 30-s delay 5-10 s	30-s delay 5-10 s vs. 30-s delay 10-15 s	30-s delay 10-15 vs 30-s delay 15-20 s	30-s delay 15-20 s vs. 30-s delay 20-25 s	30-s delay 10-25 s vs. 30-s delay 25-30 s
r value	-0.30	0.51	0.80	0.70	0.76
p value	0.0059	6.78E-07	1.15E-19	1.26E-13	8.30E-17

Treadmill off

Correlation between normalized rates, across pairs of adjacent 5-s segments in 30-s delay interval

	30-s delay 0-5 s vs. 30-s delay 5-10 s	30-s delay 5-10 s vs. 30-s delay 10-15 s	30-s delay 10-15 vs 30-s delay 15-20 s	30-s delay 15-20 s vs. 30-s delay 20-25 s	30-s delay 10-25 s vs. 30-s delay 25-30 s
r value	-0.46	0.43	0.56	0.64	0.46
p value	5.05E-05	1.33E-04	2.02E-07	1.52E-09	4.71E-05

Pearson’s linear correlation test statistics are two-sided without post-hoc adjustments for multiple comparisons. Source data are provided as a Source Data file.

Table S2. Percent delay-active cells, time cells, time-limited cells, and persistently active cells in each treadmill/delay condition, statistics by session.

	on/10-s	on/30-s	off/10-s	off/30-s
**Percent delay-active cells**				
Training and recording with treadmill on and off (N = 18 sessions)				
Mean ± SEM	32.28 ± 3.23	31.22 ± 4.08	34.6 ± 4.23	34.64 ± 4.88
ANOVA	F(3,68) = 0.18, p = 0.91			
Training and recording with only treadmill on (N = 16 sessions)				
Mean ± SEM	45.15 ± 3.77	37.59 ± 4.02		
	Training*Duration: F(1,64) = 0.782, p = 0.38;<br>Training: F(1,64) = 6.844, p = 0.011; Duration: F(1,64) = 1.374, p = 0.245			
Training and recording with only treadmill off (N = 25 sessions)				
Mean ± SEM			34.83 ± 3.48	35.95 ± 3.39
ANOVA	Training*Duration: F(1,82) = 0.02, p = 0.888;<br>Training: F(1,82) = 0.04, p = 0.843; Duration: F(1,82) = 0.023, p = 0.881			
**Percent time cells**				
Training and recording with treadmill on and off (N = 18 sessions)				
Mean ± SEM	20.05 ± 2.51	15.87 ± 2.65	18.87 ± 3.05	15.96 ± 2.04
ANOVA	F(3,68) = 0.7, p = 0.56			
Training and recording with only treadmill on (N = 16 sessions)				
Mean ± SEM	13.41 ± 2.7	10.86 ± 2.75		
ANOVA	Training*Duration: F(1,64) = 0.10, p = 0.752;<br>Training: F(1,64) = 5.097, p = 0.027; Duration: F(1,64) = 1.705, p = 0.196			
Training and recording with only treadmill off (N = 25 sessions)				
Mean ± SEM			15.75 ± 2.53	11.52 ± 2.32
ANOVA	Training*Duration: F(1,82) = 0.07, p = 0.792;<br>Training: F(1,82) = 2.298, p = 0.133; Duration: F(1,82) = 2.047, p = 0.156			
**Percent time-limited cells**				
Training and recording with treadmill on and off (N = 18 sessions)				
Mean ± SEM	11.18 ± 2.19	10.74 ± 1.89	9.09 ± 2.40	12.24 ± 1.92
ANOVA	F(3,68) = 0.41, p = 0.75			
Training and recording with only treadmill on (N = 16 sessions)				
Mean ± SEM	6.17 ± 2.21	6.74 ± 2.16		
ANOVA	Training*Duration: F(1,64) = 0.061, p = 0.806;<br>Training: F(1,64) = 4.818, p = 0.032; Duration: F(1,64) = 0.001, p = 0.976			
Training and recording with only treadmill off (N = 25 sessions)				
Mean ± SEM			10.76 ± 2.07	10.23 ± 2.24
ANOVA	Training*Duration: F(1,82) = 0.723, p = 0.398;<br>Training: F(1,82) = 0.006, p = 0.938; Duration: F(1,82) = 0.366, p = 0.547			

**Percent persistently active cells**				
Training and recording with treadmill on and off (N = 18 sessions)				
Mean ± SEM	8.87 ± 1.78	5.13 ± 1.28	9.78 ± 2.50	3.72 ± 0.93
ANOVA	F(3,68) = 3.01, p = 0.04			
Training and recording with only treadmill on (N = 16 sessions)				
Mean ± SEM	7.24 ± 1.96	4.12 ± 2.12		
ANOVA	Training*Duration: F(1,64) = 0.032, p = 0.859; Training: F(1,64) = 0.575, p = 0.451; Duration: F(1,64) = 3.901, p = 0.053			
Training and recording with only treadmill off (N = 25 sessions)				
Mean ± SEM			4.99 ± 1.53	1.29 ± 0.69
ANOVA	Training*Duration: F(1,82) = 0.653, p = 0.421; Training: F(1,82) = 6.091, p = 0.016; Duration: F(1,82) = 11.115, p = 0.001			
**Percent time-limited cells with peak within first 5 s of delay interval**				
Training and recording with treadmill on and off (N = 18 sessions)				
Mean ± SEM	98.46 ± 1.60	84.88 ± 7.92	100.00 ± 0.00	82.75 ± 8.54
ANOVA	F(3,54) = 2.15, p = 0.10			
Training and recording with only treadmill on (N = 16 sessions)				
Mean ± SEM	95.83 ± 4.45	85 ± 13.25		
ANOVA	Training*Duration: F(1,39) = 0.036, p = 0.851; Training: F(1,39) = 2.841, p = 0.10; Duration: F(1,39) = 0.03, p = 0.863			
Training and recording with only treadmill off (N = 25 sessions)				
Mean ± SEM			94.12 ± 6.06	100.00 ± 0.00
ANOVA	Training*Duration: F(1,59) = 4.401, p = 0.04; Training: F(1,59) = 1.063, p = 0.307; Duration: F(1,59) = 1.063, p = 0.307			

All statistical testing was performed using ANOVAs, which inherently do not specify a direction for the difference between means and are adjusted for multiple comparisons. Source data are provided as a Source Data file.

Table S3. Percent delay-active cells, time cells, time-limited cells, and persistently active cells in each treadmill/delay condition, statistics by animal.

	on/10-s	on/30-s	off/10-s	off/30-s
**Percent delay-active cells**				
Training and recording with treadmill on and off (N = 5 animals)				
Mean ± SEM	32.93 ± 3.39	30.58 ± 5.15	35.61 ± 5.07	35.93 ± 2.98
ANOVA	F(3,16) = 0.35, p = 0.79			
**Percent time cells**				
Training and recording with treadmill on and off (N = 5 animals)				
Mean ± SEM	20.07 ± 3.38	14.99 ± 4.37	19.07 ± 1.87	16.42 ± 1.87
ANOVA	F(3,16) = 0.58, p = 0.63			
**Percent time-limited cells**				
Training and recording with treadmill on and off (N = 5 animals)				
Mean ± SEM	11.25 ± 2.44	10.51 ± 2.98	8.35 ± 2.89	12.51 ± 1.42
ANOVA	F(3,16) = 0.48, p = 0.70			
**Percent persistently active cells**				
Training and recording with treadmill on and off (N = 5 animals)				
Mean ± SEM	8.82 ± 1.27	4.48 ± 1.61	10.72 ± 2.83	3.91 ± 0.47
ANOVA	F(3,16) = 3.56, p = 0.04			
**Percent of time-limited cells with peak in first 5 s of delay interval**				
Training and recording with treadmill on and off (N = 5 animals)				
Mean ± SEM	98.75 ± 1.25	90.18 ± 4.48	100 ± 0.0	84.78 ± 4.86
ANOVA	F(3,16) = 4.48, p = 0.02			
**Percent time cells, using the selection criteria in ref. 8**				
Training and recording with treadmill on and off (N = 5 animals)				
Mean ± SEM	4.94 ± 1.90	5.80 ± 1.91	5.35 ± 1.11	7.71 ± 1.80
ANOVA	F(3,16) = 0.51, p = 0.68			
**Percent of time cells, using the selection criteria in ref. 8, with peak in first 5 s of delay interval**				
Training and recording with treadmill on and off (N = 5 animals)				
Mean ± SEM	100.0	96.3	100.0	84.85
ANOVA	F(3,14) = 1.17, p = 0.36			

All statistical testing was performed using ANOVAs, which inherently do not specify a direction for the difference between means and are adjusted for multiple comparisons. Source data are provided as a Source Data file.

Table S4. Assembly numbers and turn-selective assemblies in the delay and stem area

		Delay				Stem			
		# of assemblies				# of assemblies			
		active[1]	turn-selective[2]	% selective	P[3]	active[1]	turn-selective[2]	% selective	P[3]
on only	on/10-s	30	8	26.7	0.0001	11	2	18.2	0.10
	on/30-s	33	3	9.1	0.23	13	0	0.00	0.51
off only	off/10-s	40	4	10.0	0.14	12	0	0.00	0.54
	off/30-s	50	5	10.0	0.10	11	0	0.00	0.57
on and off	on/10-s	61	4	6.6	0.36	24	2	8.3	0.34
	on/30-s	64	4	6.3	0.40	24	3	12.5	0.12
	off/10-s	95	8	8.4	0.10	27	6	22.2	0.0019
	off/30-s	105	9	8.6	0.080	30	10	33.3	0

[1] active in at least 4 of 20 trials, assembles were initially detected during the delay but could also activate (strength >1 standard deviation above mean) in other maze segments, [2] L/R assembly strength difference > 95% of shuffle, [3] Binomial test.
