# Supplementary material for: Time cell sequences during delay intervals are not dependent on brain state and do not support hippocampus-dependent working memory
Source: Nat Commun. 2025 Aug 12;16:7470. doi: 10.1038/s41467-025-62498-z (PMC12343883; doi:10.1038/s41467-025-62498-z)
Supplement: Supplementary file 2 — Reporting Summary [file 41467_2025_62498_MOESM2_ESM.pdf]

nature portfolio

Corresponding author(s): Stefan Leutgeb, Jill K. Leutgeb

Last updated by author(s): Jul 7, 2025

Reporting Summary

Nature Portfolio wishes to improve the reproducibility of the work that we publish. This form provides structure for consistency and transparency in reporting. For further information on Nature Portfolio policies, see our Editorial Policies and the Editorial Policy Checklist.

Statistics

For all statistical analyses, confirm that the following items are present in the figure legend, table legend, main text, or Methods section.

n/a	Confirmed	
☐	☒	The exact sample size (*n*) for each experimental group/condition, given as a discrete number and unit of measurement
☐	☒	A statement on whether measurements were taken from distinct samples or whether the same sample was measured repeatedly
☐	☒	The statistical test(s) used AND whether they are one- or two-sided<br>*Only common tests should be described solely by name; describe more complex techniques in the Methods section.*
☒	☐	A description of all covariates tested
☐	☒	A description of any assumptions or corrections, such as tests of normality and adjustment for multiple comparisons
☐	☒	A full description of the statistical parameters including central tendency (e.g. means) or other basic estimates (e.g. regression coefficient) AND variation (e.g. standard deviation) or associated estimates of uncertainty (e.g. confidence intervals)
☐	☒	For null hypothesis testing, the test statistic (e.g. *F*, *t*, *r*) with confidence intervals, effect sizes, degrees of freedom and *P* value noted<br>*Give P values as exact values whenever suitable.*
☒	☐	For Bayesian analysis, information on the choice of priors and Markov chain Monte Carlo settings
☐	☒	For hierarchical and complex designs, identification of the appropriate level for tests and full reporting of outcomes
☐	☒	Estimates of effect sizes (e.g. Cohen's *d*, Pearson's *r*), indicating how they were calculated

Our web collection on statistics for biologists contains articles on many of the points above.

Software and code

Policy information about availability of computer code

Data collection	Cheetah (Neuralynx), Version v5.6.3<br>SpikeGLX, Version 20201103
Data analysis	MATLAB (MathWorks), Version r2020a<br>Custom code, https://github.com/LiYuan-001/Time-cell-sequences-during-delay-intervals-are-not-dependent-on-brain-states

For manuscripts utilizing custom algorithms or software that are central to the research but not yet described in published literature, software must be made available to editors and reviewers. We strongly encourage code deposition in a community repository (e.g. GitHub). See the Nature Portfolio guidelines for submitting code & software for further information.

Data

Policy information about availability of data

All manuscripts must include a data availability statement. This statement should provide the following information, where applicable:

- Accession codes, unique identifiers, or web links for publicly available datasets
- A description of any restrictions on data availability
- For clinical datasets or third party data, please ensure that the statement adheres to our policy

All the data reported in this paper will be shared by the corresponding authors upon request

Research involving human participants, their data, or biological material

Policy information about studies with human participants or human data. See also policy information about sex, gender (identity/presentation), and sexual orientation and race, ethnicity and racism.

Reporting on sex and gender	N/A
Reporting on race, ethnicity, or other socially relevant groupings	N/A
Population characteristics	N/A
Recruitment	N/A
Ethics oversight	N/A

Note that full information on the approval of the study protocol must also be provided in the manuscript.

Field-specific reporting

Please select the one below that is the best fit for your research. If you are not sure, read the appropriate sections before making your selection.

☒ Life sciences ☐ Behavioural & social sciences ☐ Ecological, evolutionary & environmental sciences

For a reference copy of the document with all sections, see nature.com/documents/nr-reporting-summary-flat.pdf

Life sciences study design

All studies must disclose on these points even when the disclosure is negative.

Sample size	Sample sizes were determined based on previous electrophysiological recording studies in freely behaving rats. From all subjects, all cells with stable recordings throughout the sessions were identified and used for further analysis.
Data exclusions	Cells with very low firing rate (< 0.1 Hz) were excluded because they are unsuitable for spike-train analysis. Data from putative interneurons were excluded from some analyses.
Replication	The results with the combined treadmill on/off design were largely replicated in designs using only one of the two conditions. In addition, key results were also replicated in an additional group with Neuropixels rather than tetrode recordings.
Randomization	All animals within an experimental group were subject to the same behavioral design. Animals that were purchased from approved vendors were assigned to an experimental group without any selection criteria.
Blinding	The reported neuronal firing patterns can only be observed after extensive data analysis, and the experimenter was therefore blind to the outcome at the time of data acquisition.

Reporting for specific materials, systems and methods

We require information from authors about some types of materials, experimental systems and methods used in many studies. Here, indicate whether each material, system or method listed is relevant to your study. If you are not sure if a list item applies to your research, read the appropriate section before selecting a response.

Materials & experimental systems

n/a	Involved in the study	
☒	☐	Antibodies
☒	☐	Eukaryotic cell lines
☒	☐	Palaeontology and archaeology
☐	☒	Animals and other organisms
☒	☐	Clinical data
☒	☐	Dual use research of concern
☒	☐	Plants

Methods

n/a	Involved in the study	
☒	☐	ChIP-seq
☒	☐	Flow cytometry
☒	☐	MRI-based neuroimaging

Animals and other research organisms

Policy information about studies involving animals; ARRIVE guidelines recommended for reporting animal research, and Sex and Gender in Research

Laboratory animals	Wild-type Long-Evans rats were purchased from an approved vendor (Envigo) and were 4-7 months old for all experiments.
Wild animals	The study did not involve wild animals.
Reporting on sex	An approximately equal number of male and female rats were used in all experiments, with the exact numbers provided in the Methods section. The study was not powered to perform sex-based analyses, and all analyses are therefore performed on aggregate data.
Field-collected samples	The study did not involve samples collected from the field.
Ethics oversight	The animal protocol (S08276) was approved by the Institutional Animal Care and Use Committee of the University of California, San Diego

Note that full information on the approval of the study protocol must also be provided in the manuscript.

Plants

Seed stocks	*Report on the source of all seed stocks or other plant material used. If applicable, state the seed stock centre and catalogue number. If plant specimens were collected from the field, describe the collection location, date and sampling procedures.*
Novel plant genotypes	*Describe the methods by which all novel plant genotypes were produced. This includes those generated by transgenic approaches, gene editing, chemical/radiation-based mutagenesis and hybridization. For transgenic lines, describe the transformation method, the number of independent lines analyzed and the generation upon which experiments were performed. For gene-edited lines, describe the editor used, the endogenous sequence targeted for editing, the targeting guide RNA sequence (if applicable) and how the editor was applied.*
Authentication	*Describe any authentication procedures for each seed stock used or novel genotype generated. Describe any experiments used to assess the effect of a mutation and, where applicable, how potential secondary effects (e.g. second site T-DNA insertions, mosiacism, off-target gene editing) were examined.*
